# miR-30a-5p suppresses lung squamous cell carcinoma via ATG5 - mediated autophagy

**DOI:** 10.18632/aging.203235

**Published:** 2021-07-12

**Authors:** Jichen Yang, Sunyin Rao, Run Cao, Shouyong Xiao, Xin Cui, Lianhua Ye

**Affiliations:** 1 Department of Thoracic Surgery, Lung Cancer Research Center, Yunnan Institute of Oncology, the Third Affiliated Hospital of Kunming Medical University, Yunnan Cancer Hospital, Kunming Yunnan 650118, PR China

**Keywords:** lung squamous cell carcinoma, ATG5, miR-30a-5p, autophagy, prognosis

## Abstract

Propose: Autophagy plays a complicated role in cancer progression. This study aims at assessing the function of ATG5-induced autophagy in progression of lung squamous cell carcinoma and its upstream mechanism.

Method: TCGA database of lung squamous cell carcinoma was analyzed to explore the differentially expressed miRNAs and mRNAs and relative prognosis. RT-PCR and Western blot were performed to evaluate autophagy relative gene expression level in human lung squamous cell carcinoma cell Lines. Autophagy flux was observed using transmission electron microscopy and immunofluorescence. Meanwhile, binding relationship of potential target miRNA and mRNAs were also confirmed using Dual-luciferase reporter gene assay. Lung metastatic model was established to evaluated the effect of targeting protein and miRNA.

Result: High level expression of ATG5 was detected in LUSC patients. Relative experiments confirmed that ATG5 silencing could decrease the autophagy flux in LUSC. In addition, our research revealed that there is a binding sites between hsa-mir-30a-5p and 3′-UTR of ATG5. Mimic miR-30a-5p suppresses ATG5-mediated autophagy in lung squamous cell carcinoma cells. The *in vivo* experiments confirmed that miR-30a-5p could attenuate lung squamous cell carcinoma progression through the autophagy pathway.

Conclusion: Accordingly, the *in vivo* and *in vitro* study in our research have demonstrated that miR-30a-5p inhibits lung squamous cell carcinoma progression via ATG5-mediated autophagy.

## INTRODUCTION

With high morbidity and poor prognosis, lung cancer has become the leading cancer cause of death [1]. Overall, 80–85% of all human lung cancers are non-small cell lung cancer (NSCLC) which includes two major histological subtypes: squamous cell carcinoma and adenocarcinoma [2]. Lung squamous cell carcinoma (LUSC), as a subtype of NSCLC, accounts for approximately 40% of all lung cancer, which based on age or extent of tobacco exposure [3]. Compared with lung adenocarcinoma, LUSC lacks effective therapeutic target and shows a poor clinical prognosis [4].

Macroautophagy is defined as a lysosome-conducted progress by which cells decompose protein organelles and sort of metabolic waste [5]. Former research suggests that autophagy plays a complicated role in cancer progression, which may both suppress and promote tumor functions [6, 7]. Former study has confirmed that autophagy could attenuate the initial stages of tumorigenesis by suppressing immunosuppression protein such as PD-L1 [8, 9, 10]. Meanwhile, autophagy, which was an energy-producing process, could also nurture established cancers and so on promoting cancer progression [11, 12]. Yang et al. [13] revealed that autophagy induces tumor growth by alternating p53. According to former research relating to oral squamous cell carcinoma, autophagy was considered to play a key role in either nurturing cancer cells [13] or regulating cell apoptosis [14, 15].

Autophagy-related gene 5 (ATG5), which induces autophagosome formation, forms a complex with ATG12 and ATG16L1. Then, the ATG5–ATG12–ATG6L1 complex is required to conjugate of LC3 (ATG8) family members and the lipid phosphatidylethanolamine (PE) [16, 17]. LC3 will be inserted on the emerging AV after conjugated to lipid [18, 19]. The lipidated form of LC3 (LC3-II) migrates faster than LC3-I on gel electrophoresis, allowing the ratio of lipidated to free LC3 to serve as an approximation of the number of AVs forming at any given time. According to the over-expression of ATG5 in LUSC, we suspect ATG5 may inhibit LUSC progression by autophagy pathway.

Herein, ATG5-mediated autophagy should be considered as factor which promotes cancer progress. Those evidences revealed that autophagy may be a potential target for LUSC treatment. In this study, relationship between prognosis and ATG5 expression was firstly evaluated. The function of ATG5 on autophagy in LUSC was further confirmed. Moreover, we firstly explored that hsa-miR-30a-5p could suppress LUSC cells autophagy by down regulating ATG5 expression. Therefore, exploring the molecular mechanism of ATG5 may provide a novel strategy of anti-tumor treatment for LUSC.

## MATERIALS AND METHODS

### Bioinformatics

The profiles of mRNA (Normal: 52, Tumor: 503) and miRNA (Normal:44, Tumor:336) were obtained from TCGA-LUSC clinical data (https://portal.gdc.cancer.gov/). The “edgeR” package (|logFC|>1, padj<0.05) was used to analyze differentially expressed miRNAs (DEmiRNAs) between normal group and tumor group. The potential upstream miRNAs of LUSC were predicted according to databases of miRDB and starBase. DEmiRNAs was used to explore the bind sites between the miRNAs and target mRNA. The kaplan-meier analysis was used for target mRNA and corresponding miRNAs based on TCGA-LUSC dataset.

### Cell lines and culture

Human LUSC cell lines NCI-H1730, NCI-H2170, LOU-NH91, SW900 and human alveolar epithelial cells line HPAEpic were all purchased from Bluefcell (Bluefcell, Shanghai, China). Complete medium was used for cell culture. All cells line were then nurtured according to former relative research [15].

### Cell transfection

The relative plasmids, including Mimic NC, mimic miR-30a-5p, si-NC and si-ATG5, were purchased from Genomeditech (Shanghai, China). Lipo 3000 (Thermo Fisher Scientific, Inc.) was used in plasmid transfection. The ATG5 down-regulate lentivirus were also purchased from Genomeditech and transfected into LUSC cell to obtain ATG5 low-expression LUSC cell line.

### RNA extraction and RT-PCR

Comply with manufacturer instruction, Axygen RNA Miniprep Kit (Axygen, USA) was used to obtain total RNA. The following process were performed according to former relative research [15]. The human primer sets used were as follows: GAPDH forward: 5′-CCTCTGACTTCAACAGCGAC-3′ and reverse: 5′-TCCTCTTGTGCTCTTGCTGGC-3′; ATG5 forward: 5′-TGCAGATGGACAGTTGCACA-3′ and reverse: 5′-CCACTGCAGAGGTGTTTCCA-3′.

### Western blot analysis

Proteins were extracted from HPAEpic and LUSC cells, si-nc/si-ATG5 LUSC cells and mimic nc/mimic-miR-30a-5p stimulate LUSC cells. The membranes were blocked by Quickblock (Beyotime, China) at 20°C for 20 min. Then, strip membranes for specific kda was incubated with corresponding primary antibodies (β-actin, 1:1000; ATG5, 1:1000; P62, 1:1000; LC3B II, 1:1000; Beclin1, 1:1000) at room temperature for 2 hours. The following step were performed under the instructions of manufacturer.

### Transmission electron microscopy (TEM)

After starving for 2 hours, HPAEpic and SW900 cells were fixed to obtain TEM images, the process was performed as described previously [20]. JEM 1230 transmission electron microscopy (JEOL, Akishima, Tokyo) was used to analyze cell samples at 60 kV. The micrographs were obtained at ×5000 and ×20,000 magnification.

### Autophagic flux assay

SW900 cells were seeded into 6-well dishes. 24 hours later, GFP-mRFP-LC3 construct lentivirus were transfected into SW900 cells were. The cells was starved for 2 hours to stimulate autophagy. The autophagy flux was observed using an EVOS M5000 fluorescence microscope (Thermo Fisher, USA).

### Dual-luciferase reporter gene assay

The luciferase reporter vectors, including wild-type ATG5 (ATG5-WT) and mutant-type ATG5 (ATG5-MUT) were purchased from Genomeditech (Shanghai, China). HEK293T cells and Lipo 3000 were used to co-transfected with ATG5-WT/ATG5-MUT vector, miR-30a-5p mimic/mimic NC. 48 hours later, lysates of cell were collected. Dual-luciferase reporter gene assay system (Promega) was used to evaluate the luciferase activity.

### Animal models

Twelve male 6-week-old SPF BALB/c nude mice were purchased from Kunming experimental animal center (Yunnan, China) and placed in the SPF facility of the Kunming Medical University. All laboratory procedures were approved by the laboratory animal care and use committee of the hospital. High metastatic squamous cell carcinoma was established. The WT-SW900/ siATG5-SW900/ mimic miR-30a-5p-SW900/ siATG5+mimic miR-30a-5p-SW900 cells (5 × 10^5^) were suspended in 50 μL of PBS were injected into tail intravenous respectively. The mice were divided into four different groups (3 mice in each group) and received various treatment regimens: (a) control; (b) siATG5; (c) mimic miR; (d) siATG5+mimic miR. The mice bearing tumors were sacrificed 14 days after injection.

### Immunohistochemistry

To evaluate autophagy, the tumor slices were stained using anti-LC3B I/II and anti-P62 primary antibody (dilution 1:200, CST Inc.), Alex 488-conjugated goat anti-rabbit and 555-conjugated goat anti-mouse secondary antibody (dilution 1:200, CST Inc.) following the instructions. Cell nuclei were stained with DAPI (dilution 1:5000, Invitrogen). The obtained slices were observed by a confocal microscopy (Leica SP5).

### miRNA sequencing

A panel of human LUSC cell lines was infected with si-ATG5/si-nc targeting. The miRNA sequencing was performed using the HiseqXTen platform (Genechem Co., Ltd, Shanghai, China). Fold-change values was used to evaluate change of miRNAS expression. The thresholds set for differential gene expression were a fold change ≥2.0.

### Cell proliferation assay

To assess the cytotoxicity of siATG5, SW900 and NH91 cells were seeded in 96-well dishes in triplicate with 1 × 10^3^ cells/well, transfected with si-ATG5 or si-nc. A cell proliferation assay was performed using 3-(4, 5-dimethylthiazol-2-yl)-2, 5-diphenyltetrazolium bromide (MTT) solution (0.5 mg/ml) at different time point.

### Statistical analysis

GraphPad Prism 6.0 (Graphpad Software Inc.) was used for statistical analysis. All values are presented as the mean ± standard deviation (SD). Student's *t* test was used to evaluate statistical differences. Values were determined to be significant at ^*^P < 0.05, ^**^P < 0.01 and ^***^P < 0.001.

### Ethics approval and consent to participate

The study design was approved by the Ethical Committee of Yunnan Cancer Hospital.

## RESULTS

### ATG5 expresses higher in LUSC and predicts poor prognosis

**Figure 1:**
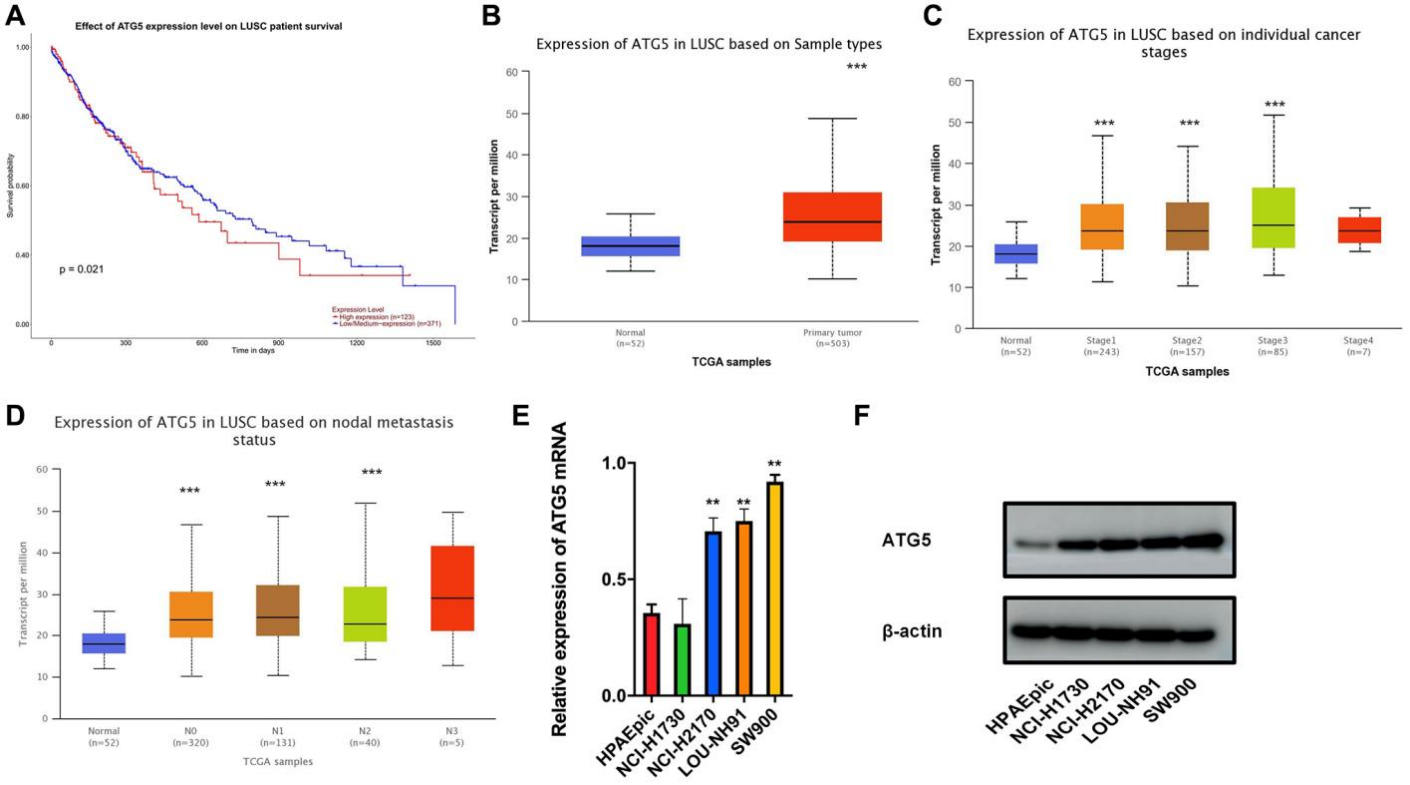
**The expression and prognosis of ATG5 in LUSC.** (**A**) Survival curves of ATG5 expression for prognosis in the TCGA-LUSC dataset. Red represents high expression group and blue was for low expression; (**B**) Compare with normal group(blue), the expression of ATG5 was high in tumor samples (red) (**C** and **D**) Box plots of ATG5 expression in different clinical stages, N stages of LUSC; (**E** and **F**) mRNA and protein expressions of ATG5 in HPAEpic and LUSC cell lines (NCI-H1730, NCI-H2170, LOU-NH91 and SW900) All the *P* values were compared with the control ^*^P < 0.05, ^**^P < 0.01, ^***^P < 0.0001.

Analysis of mRNA expression in TGCA-LUSC dataset indicates that, compare with ATG5 low-expressed patients, the survival time of high-expressed patients was lower. (Figure 1A) Based on the evaluation of TGCA-LUSC database, we confirmed that the ATG5 expression was higher in tumor tissue than normal tissue. (Figure 1B) It also shown that ATG expression increased along with stages (Figure 1C) and metastasis (Figure 1D) progression. In accordance with the TGCA database, RT-PCR and western blot results shown that LUSC cells lines express higher ATG5 than that in HPAEpic cells line. (Figure 1E, 1F).

### ATG5 down regulation inhibits autophagy in LUSC cells

**Figure 2:**
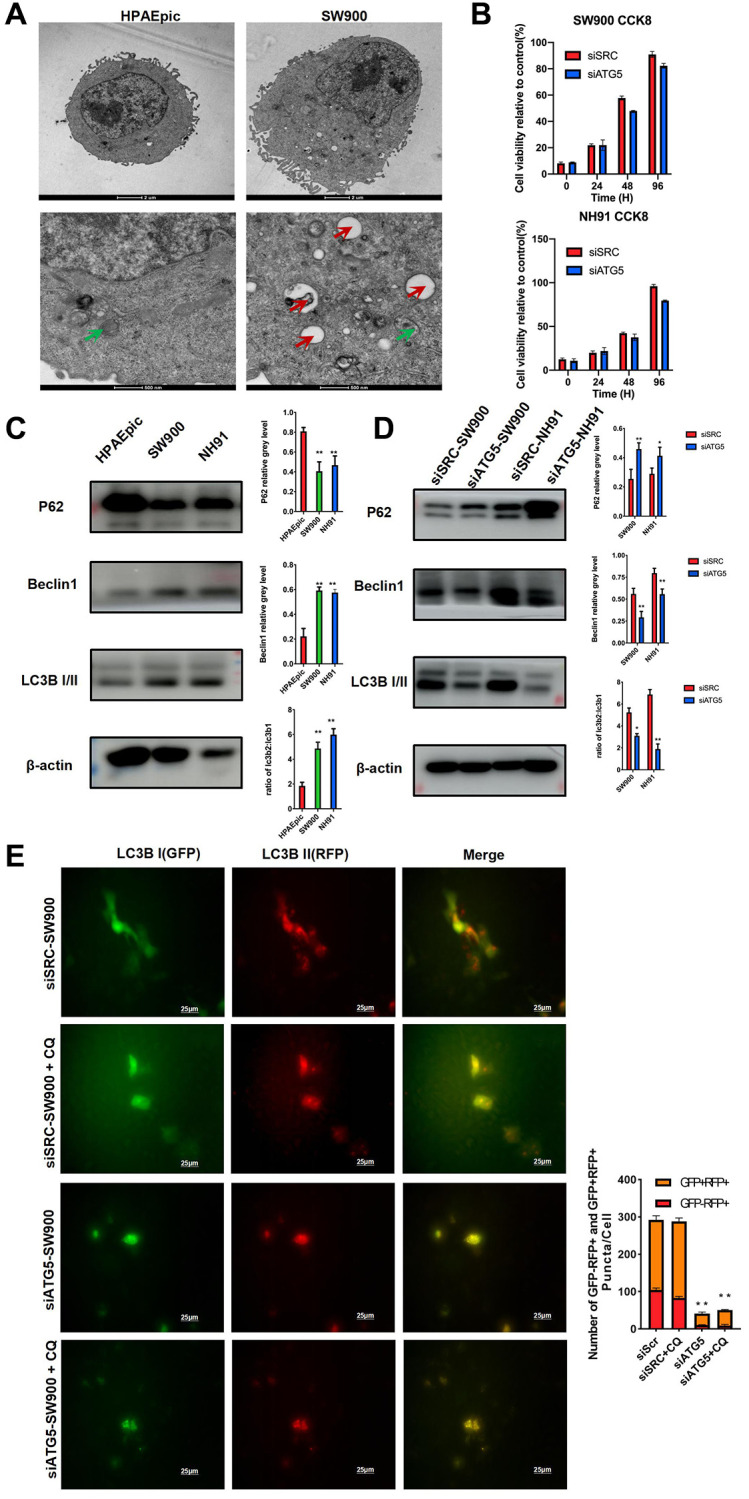
**ATG5 down regulation inhibits autophagy in LUSC cells.** (**A**) Representative TEM images showing the formation of autophagosomes (red arrow) and autolysosomes (green arrow) in HPAEpic and SW900. (**B**) After the transfected with siATG5/SRC, cell viability at different time points were assessed by using an MTT assay. (**C**) Expression of autophagy relative protein, including P62, Beclin1 and LC3B I/II, were evaluated in HPAEpic, SW900 and NH91cells. (**D**) Expression of ATG5, P62, Beclin 1, and LC3B I/II in SW900 and NH91 cells transfected with si-ATG5 were detected using a western blot assay. (**E**) siATG5 and siSRC SW900 cells were transfected with GFP-mRFP-LC3 virus and incubated with or without chloroquine for 24 h and then starved for 2 hours. Red or yellow puncta indicating complete/incomplete autophagy were calculated and reported as the mean ± SD. All the *P* values were compared with the control ^*^P < 0.05, ^**^P < 0.01, ^***^P < 0.0001.

To explore the function of ATG5 on LUSC cells, the morphology of the HPAEpic and SW900 cells was examined by transmission electron microscopy (TEM). Compare with HPAEpic, SW900 shown an activated autophagy flux by increasing the number of autophagosomes and autolysosomes. (Figure 2A) To assess the cytotoxicity of siATG5, a cell proliferation assay was performed. Although the cell viability is lower in siATG5 group, no significant difference was observed between the two different group. (Figure 2B) To further evaluate autophagy in LUSC cells, expression of autophagy relative protein was evaluated. Compare to HPAEpic cells, Beclin 1 and LC3B II expression was higher in SW900 and NH91 cells. P62, a marker effective autophagosomes degradation, expresses lower in LUSC cells. (Figure 2C) To evaluate the function of ATG5 in autophagy, the down-regulated ATG5 suppressed expression of Beclin 1 and LC3B II and up regulated P62 expression. (Figure 2D) These results demonstrate that ATG5 was necessary for process autophagy flux in LUSC. In conformity to western blot result, immunofluorescence result using GFP-mRFP-LC3B lentivirus shown an decrease autophagy flux was decreased in siATG5-affected LUSC cells. Interestingly, ratio of yellow puncta increased significantly after the autolysosome degradation was inhibited by chloroquine in both group. (Figure 2E) The yellow fluorescence puncta shown the incomplete autophagy. The red fluorescence puncta shown in merge images indicated complete autophagic flux.

### ATG5 mRNA expression is inhibited by miR-30a-5p

**Figure 3:**
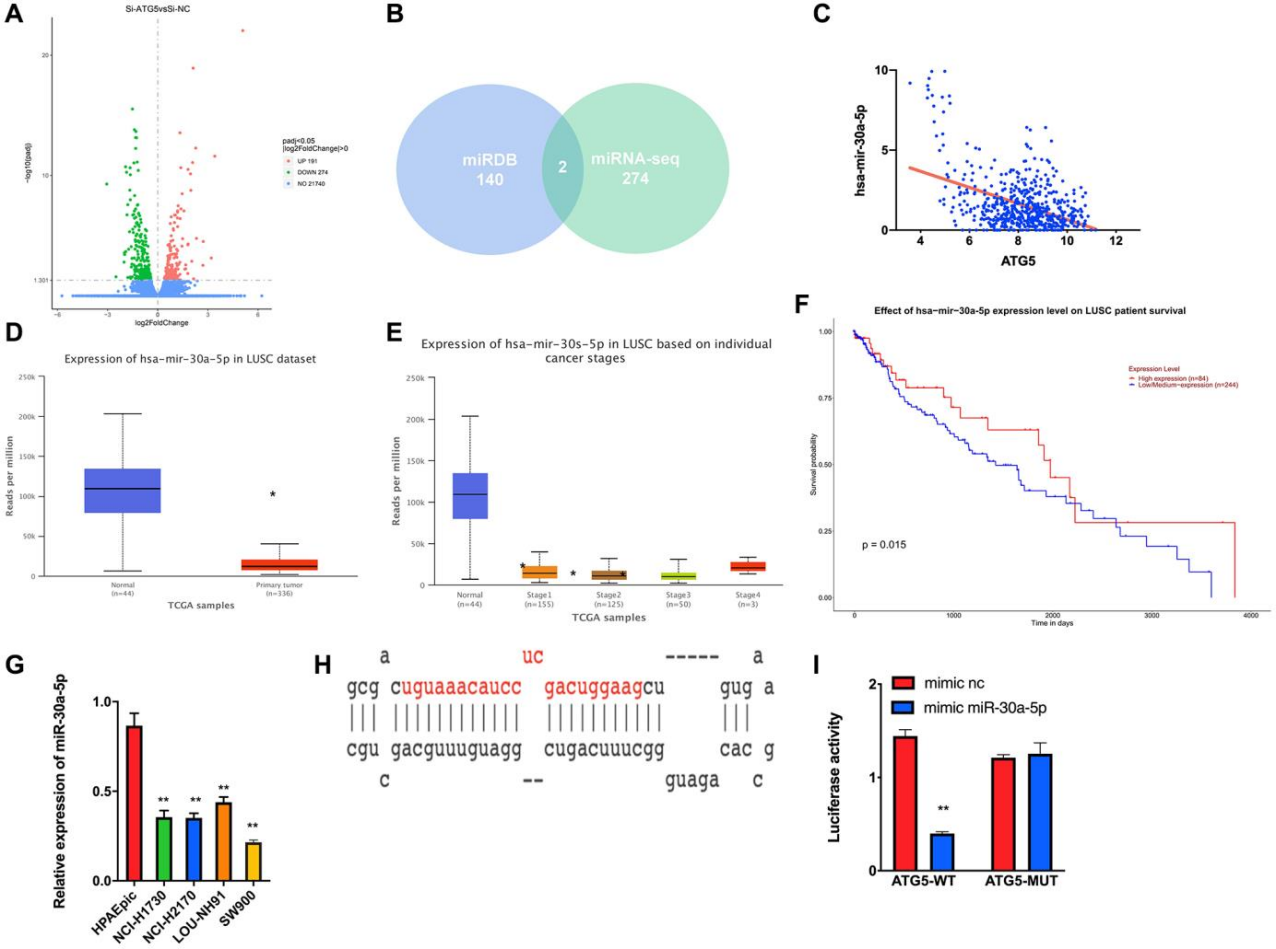
**miR-30a-5p suppresses ATG5-mediated autophagy in LUSC.** (**A**) DEmiRNA volcano map of siATG5 and siSRC SW900 group based. (**B**) Venn diagram of predicted upstream down-regulated DEmiRNAs for ATG5. (**C**) Pearson correlation analysis of ATG5 and miR-30a-5p; (**D**) The miR-30a-5p expression was down regulated in tumor group (red) compared with normal samples (blue); (**E**) Box plots of miR-30a-5p expression in different clinical stages of LUSC; (**F**) Survival curves of miR-30a-5p expression for prognosis. Red indicates high expression group and blue indicates low expression group; (**G**). Expression of miR-30a-5p in HPAEpic cell line and LUSC cell lines. (**H**) Binding sites of miR-30a-5p and 3‘UTR of ATG5; (**I**) Dual-luciferase reporter gene assay was used to determine the targeted binding of miR-30a-5p and ATG5.

The miRNA sequencing was performed to analyze ATG5 upstream regulatory pathway was conducted. To begin with, a total of 274 down-regulation miRNAs were obtained (Figure 3A). Meanwhile, the potential miRNAs regulating ATG5 was predicted by using miRDB databases. From 274 down-regulated miRNAS, miR-30a-5p was obtained by cross check (Figure 3B). A negative correlation between ATG5 and miR-30a-5p was determined using Pearson correlation analysis (Figure 3C). According to TCGA-GA database, low-expressed of miR-30a-5p in tumor tissues was observed (Figure 3D, 3E), which also indicates a poor prognosis (Figure 3F) in LUSC. RT-PCR results also confirmed that expression of miR-30a-5p is lower in LUSC cells than in HPAEpic (Figure 3G). Then, miRDB and starBase were used to explore the binding regions between ATG5 mRNA and miR-30a-5p (Figure 3H). According to the Dual-luciferase reporter gene assay result, miR-30a-5p effectively down regulates luciferase activity in ATG5-WT cell (Figure 3I).

### miR-30a-5p suppresses ATG5-mediated autophagy in LUSC

**Figure 4:**
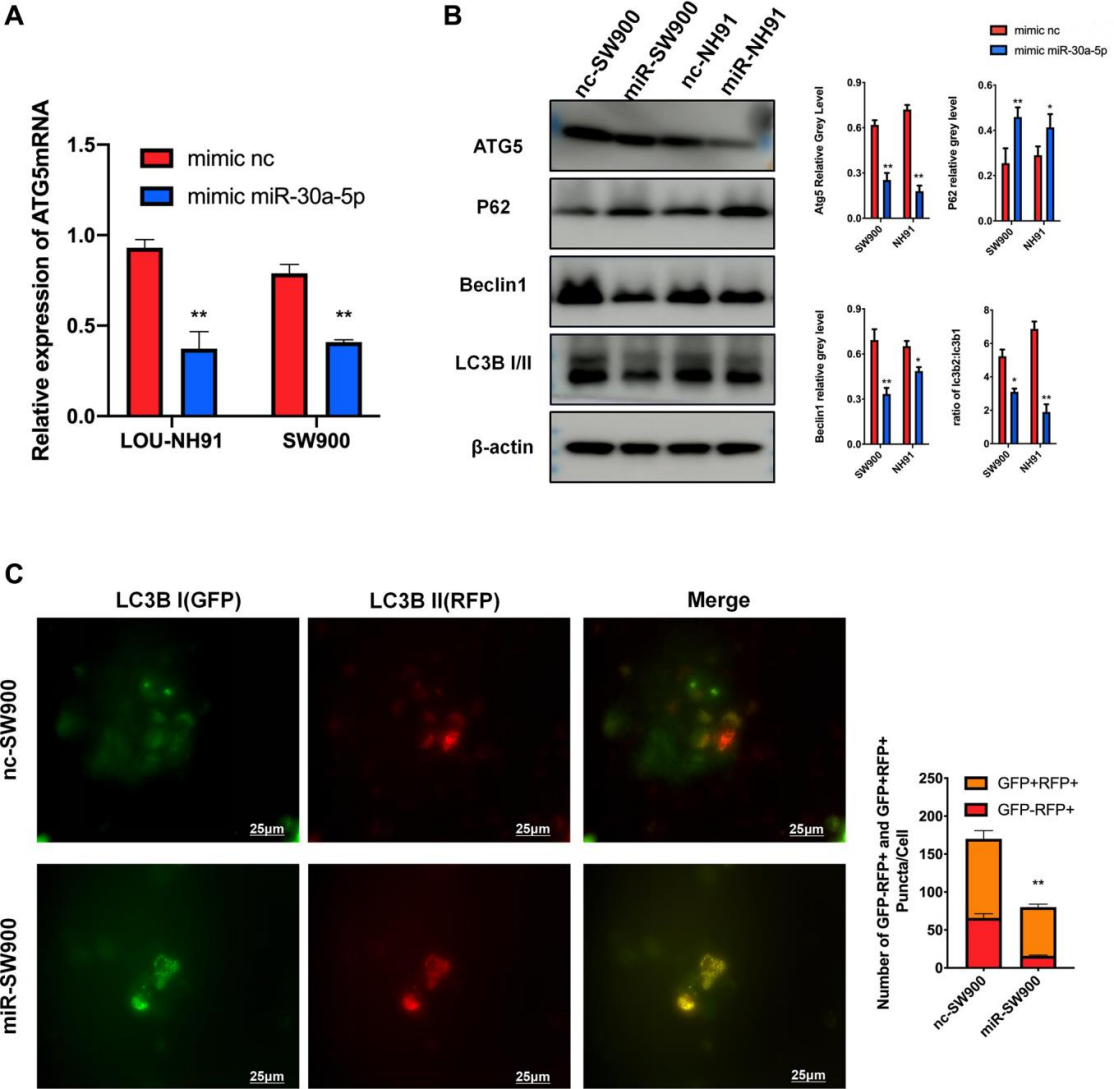
**miR-30a-5p suppresses ATG5-mediated autophagy in LUSC.** (**A**) The result of mRNA were used to evaluate the effect of miR-30a-5p on ATG5. (**B**) SW900 and NH91 cells were transfected with mimic NC or mimic miR-30a-5p and then starved for 2 hours to stimulate autophagy. ATG5, P62, Beclin 1, and LC3B I/II expression were evaluated by western blot. (**C**) GFP-mRFP-LC3 lentivirus was used to evaluate autophagy flux in mimic NC/miR transfected SW900 cells. Red or yellow puncta indicating complete/incomplete autophagy were calculated and reported as the mean ± SD. All the *P* values were compared with the control ^*^P < 0.05, ^**^P < 0.01, ^***^P < 0.0001.

According to RT-PCR results, mRNA expression of ATG5 decreased significantly under the treatment of miR-30a-5p mimic (Figure 4A). Meanwhile, miR-30a-5p induced ATG5 suppression effectively decreased ATG5, Beclin 1 and LC3B II expression, which represent the down-regulation of autophagy flux (Figure 4B). The increased P62 protein levels, which represents block of autophagolysosome degradation, was also observed (Figure 4B). The immunofluorescence results (Figure 4C) show an decrease of red puncta in miR-SW900 group, which is same with siATG5 experiment. Interestingly, an increase number of the yellow puncta, which represents impaired autophagy, was observed in miR-SW900 group.

### Attenuation of miR-30a-5p/ATG5-regulated autophagy inhibits LUSC progression in Lung metastatic model

**Figure 5:**
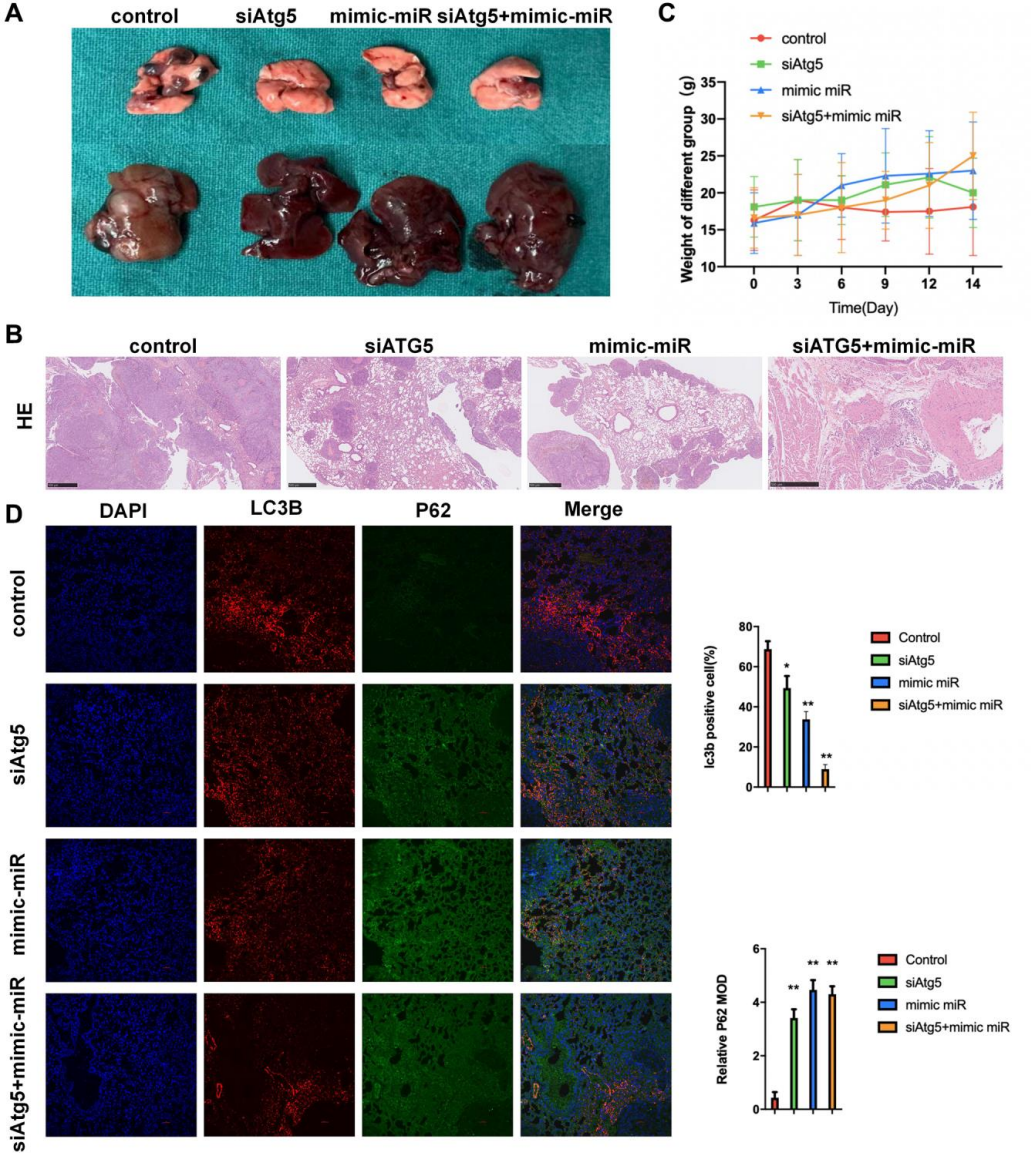
**Attenuation of miR-30a-5p/ATG5-regulated autophagy inhibits LUSC progression in lung metastatic model.** (**A**) Mice were administered WT/siATG5/mimic miR-30a-5p/siATG5+mimic miR-30a-5p transfected SW900 cells (5 × 10^5^/mouse) by i.p injection. Image representative gross appearance of lungs and livers from control (*n* = 3), siATG5 (*n* = 3), mimic miR-30a-5p (*n* = 3) and siATG5+mimic miR-30a-5p (*n* = 3) mice. (**B**) HE staining images of pulmonary metastatic samples in different group. (**C**) The body weight of mice in different groups. (**D**) Representative images of tumors from lung using immunofluorescence staining against DAPI P62, and LC3B. All the *P* values were compared with the control. ^*^P < 0.05, ^**^P < 0.01.

To evaluate the functions of miR-30a-5p/ATG5-regulated autophagy on LUSC *in vivo*, we first established a classical lung metastatic model using a previously established protocol. Compared with the WT counterparts, mice bearing siATG5-SW900 tumor or mimic miR-30a-5p-SW900 had less LUSC incidence, smaller histological lesions and less liver metastases (Figure 5A, 5B). According to Figure 5C, no statistical difference of body weight was observed among siATG5, mimic miR and siATG5+mimic miR groups. Meanwhile, significant difference was confirmed between control and siATG5+mimic miR groups.The cell nuclei, autophagy flux and autophagolysosome degradation were stained with DAPI (blue), anti-LC3B antibody (red), anti-P62 antibody(green), respectively. Compared with the control group, tumor slices from siATG5+mimic miR-SW900 bearing mice showed obviously increased green fluorescence, which indicates an accumulation of P62 and low level of autophagolysosome degradation. The reduced green fluorescence indicates an decrease of LC3B and low level of autophagy flux (Figure 5D).

## DISCUSSION

In this study, we demonstrated increased autophagy flux induced by over-expression of ATG5 in LUSC, which was regarded as an cancer promoting elements. Further research of mechanism revealed that ATG5 mRNA expression was down regulated by miR-30a-5p. Autophagy, as an double-edge sword in tumor progression, can both promote and inhibit tumor growth and the roles of autophagy may vary in different types of cancer [21]. On the one hand, autophagy can function as a tumor suppressive mechanism by inhibiting oncogenic protein accumulation, preventing tumor initiation [22, 23]. On the other hand, at the late stage of tumor progress, autophagy can nurture cancer cell, and then maintain tumor metabolism, growth, finally induces tumorigenesis and causes resistance to therapeutic agents [22, 24].

A cluster of ATGs is indispensable in autophagosome formation and relative protein degradation. Previous study have shown that down-regulated ATGs, including ATG4(also know as LC3B) and ATG7, synergized the anti-cancer function of chemotherapy [25, 26]. Accordingly, ATG5-silence mediate autophagy suppression could enhance effect of anti-tumor therapy on advanced solid cancer. Based on our research, down-regulated ATG5 effectively attenuated autophagy flux via the classic LC3B pathway. Meanwhile, immunofluorescence image shown that the silencing of ATG5 block autophagy flux *in vitro*, providing the potential strategy of LUSC treatment by chemotherapy combined with ATG5-targeted inhibitor.

According to former study relative to miRNAs in cancer, miR-30a plays an important role in tumor progress. [27] Previous study have shown the function of miR-30a-5p on autophagy. Xu et al. revealed that miR-30a increases radiosensitivity of prostate cancer by inhibiting autophagy [28]. Lin et al. suggested miR-30a sensitized LUSC against chemotherapy via autophagy suppression [29]. However, the mechanism of how miR-30a-5p inhibits autophagy in LUSC is still unclear. In Figure 3, we firstly explored the regulation of miR-30a-5p on ATG5 mRNA by Dual-luciferase reporter gene assay, which explains the potential mechanism of miR-30a-5p - mediated autophagy.

Interestingly, the immunofluorescence image of Figure 3C demonstrated that use of miR-30a-5p could not only down regulate autophagy flux but also impaired autophagy, effect of which is different from function of siATG5. Those results indicates that miR-30a-5p may have other influence on autophagy signaling except regulating ATG5. Accordingly, the *in vivo* and *in vitro* study in our research have demonstrated the suppression of miR-30a-5p on LUSC progression via ATG5-mediated autophagy. This discover may provide a new strategy for combined chemo- or biotarget- therapy in the future and hopefully improve prognosis of patients with high-express ATG5 LUSC.
